# Genetic alterations of IDH1 and Vegf in brain tumors

**DOI:** 10.1002/brb3.718

**Published:** 2017-08-01

**Authors:** Silvia Veganzones, Virginia de la Orden, Lucía Requejo, Beatriz Mediero, María Luisa González, Náyade del Prado, Carmen Rodríguez García, Raquel Gutiérrez‐González, Alvaro Pérez‐Zamarrón, Armando Martínez, Marisa L. Maestro, Horacio Mario Zimman, Anna González‐Neira, Jesús Vaquero, Gregorio Rodríguez‐Boto

**Affiliations:** ^1^ Department of Clinical Analysis Hospital Clínico San Carlos and Hospital Universitario Puerta de Hierro‐Majadahonda Majadahonda Madrid Spain; ^2^ Department of Neurosurgery Hospital Clínico San Carlos and Hospital Universitario Puerta de Hierro‐Majadahonda Majadahonda Madrid Spain; ^3^ Department of Epidemiology Hospital Clínico San Carlos and Hospital Universitario Puerta de Hierro‐Majadahonda Majadahonda Madrid Spain; ^4^ Department of Pathology Hospital Clínico San Carlos and Hospital Universitario Puerta de Hierro‐Majadahonda Majadahonda Madrid Spain; ^5^ Department of Surgery Faculty of Medicine Universidad Complutense and Universidad Autónoma Madrid Spain; ^6^ Present address: Hospital Universitario Puerta de Hierro‐Majadahonda c/ Manuel de Falla, 1 28222 Majadahonda Madrid Spain

**Keywords:** brain tumors, glioma, *IDH1*, survival, *VEGF*

## Abstract

**Background:**

This study evaluates the presence of R132H mutation in isocitrate dehydrogenase (*IDH1*) gene and the vascular endothelial growth factor (*VEGF*) +936 C/T polymorphism in brain tumors. The impact of these genetic alterations on overall survival (OS) and progression free survival (PFS) was evaluated.

**Methods:**

A cohort of 80 patients surgically treated at Hospital Clínico San Carlos, Madrid, between March 2004 and November 2012, was analyzed. Tumors were distributed in 73 primary brain tumors (gliomas, meningiomas, hemangiopericytomas and hemangioblastomas) and seven secondary tumors evolved from a low grade glioma, thus providing a mixed sample.

**Results:**

*IDH1* ^*R132H*^ gene mutation was found in 12 patients (15%) and appears more frequently in secondary tumors (5 (71.4%) whereas in 7 (9.7%) primary tumors (*p* < .001)). The mutation is related to WHO grade II in primary tumors and a supratentorial location in secondary tumors. The OS analysis for *IDH1* showed a tendency towards a better prognosis of the tumors containing the mutation (*p* = .059).The *IDH1* ^*R132H*^ mutation confers a better PFS (*p* = .025) on primary tumors. The T allele of *VEFG* +936 C/T polymorphism was found in 16 patients (20%). No relation was found between this polymorphism and primary or secondary tumor, neither with OS or PFS.

**Conclusions:**

*IDH1* ^*R132H*^ gene mutation is exclusive in supratentorial tumors and more frequent in secondary ones, with a greater survival trend and better PFS in patients who carry it. The T allele of *VEGF* +936 C/T polymorphism is more common in primary tumors, although there is no statistical relation with survival.

## 1 Introduction

Primary brain tumors account for 85%–90% of all primary central nervous system tumors (Mehta, Vogelbaum, Chang, & Patel, 2011), with an estimate prevalence and mortality of 1.8% and 2.3% of all cancers respectively (Ferlay et al., 2015). The most frequent brain tumors are gliomas, which represent 50% of Central Nervous System tumors.

According to the World Health Organization classification, there are three types of gliomas: astrocytomas, oligodendrogliomas (ODGs) and ependymomas. There are four grades of astrocytomas (I to IV) based on nuclear atypia, mitosis, vascularization and necrosis. ODGs and ependymomas are classified in two grades: low grade and anaplastic (Ducray et al., 2011). These tumors are classified as primary brain tumors, when there is no prior low‐grade tumor and secondary brain tumors, when they evolve from a glioma of a lower histological grade. Two major characteristics of gliomas are the tendency to progression to more malignant variants and the invasion of deep brain structures. Glioblastoma multiform (GBM), classified as a grade IV astrocytoma, is the most malignant and common brain tumor in adults, representing 50% of all gliomas. Median overall survival of gliomas is 13.9 months, with 2 years survival of 22.5% and 5 year survival of 5.3% (Brennan et al., 2013). The median survival rate for patients with GMBs is 12.1–14.6 months, although 2–5% of patients are long‐term survivors (more than 3 years; Bleeker, Molenaar, & Leenstra, 2012; Polivka et al., 2014). For lower grades, the survival rate is between 4 and 15 years. Malignant meningiomas have a 5 years survival of 65% (Ostrom et al., 2014).

Neovasculogenesis and metabolic alterations drive the growth and self‐renewal of gliomas, thus leading to tumor progression due to the lack of control of tumor cells. Over the last years, genetic studies have been promoted over the last years and new molecular markers have appeared offering useful information in diagnosis, prognosis and target therapies for individual patients. The management of the intracellular oxidative damage is an important mechanism for cells survival which is regulated through the production of cytoplasmic nicotinamide adenine dinucleotide (NADPH). Low levels of NADPH result in a reduction of glutathione (GSH) that is the most abundant intracellular antioxidant involved in the protection of cells (Shi et al., 2015). The Isocitrate Dehydrogenase‐1 protein (IDH1) plays a significant role in this process. *IDH1* mutated cells present decreased levels of NADPH and GSH compared to wild‐type *IDH1* and control cells (Shi et al., 2015). *IDH1* gene is located on 2q33.3, and encodes a cytosolic NADPH –IDH1‐dependent enzyme (Thota et al., 2012). The IDH1 enzyme catalyzes the oxidative decarboxylation of isocitrate, producing alpha‐ketoglutarate (α‐KG), reducing NADP+ to NADPH. NADPH is essential for regenerating reduced GSH, which is important in protecting cells against oxidative stress (Ducray et al., 2011). Mutated enzyme IDH1 catalyzes the reduction in α‐KG to NADP‐dependent R2‐hydroxyglutarate (R2‐HG), which causes a reduction in GSH and in prolyl oxidases levels and promotes the accumulation of hypoxia inducible factor (HIF‐1α; Ducray et al., 2011; Parsons et al., 2008; Thota et al., 2012).

Parsons et al. (2008) first described the relation between *IDH1* gene mutation in 12% young patients harboring GBM and improved clinical prognosis. Most of *IDH1* mutations in glioma tumors are heterozygous point mutations in codon 132. Among these, more than 90% affect the amino acid arginine at position 132, converting it to histidine (R132H; Lewandowska et al., 2014; Thota et al., 2012). *IDH1* ^*R132H*^ mutation overexpression has been shown to decrease cell proliferation (Parsons et al., 2008). Several studies show the presence of the *IDH1* ^*R132H*^ mutation in 70–90% of secondary GBMs and 10% primary GBMs, both cases associated with young patients and a higher overall survival rate (Bleeker et al., 2012; Carrillo et al., 2012; Mellai et al., 2011; Weller et al., 2009). The median survival of patients with *IDH1* mutation is 3.8 years compared with 1.1 years in patients with wild‐type *IDH1* (Parsons et al., 2008).

Angiogenesis contributes to the development, growth and progression of solid tumors (Yancopoulos et al., 2000). The vascular endothelial growth factor (VEGF) is one of the most potent endothelial cell mitogens and plays an important role in angiogenesis (Roy, Bhardawaj, & Ylä‐Herttuala, 2006). The *VEGF* gene is located on chromosome 6p21.3 and more than 30 single‐nucleotide polymorphisms (SNPs) have been identified in this gene (Ku et al., 2005; Lin, Wu, Tsai, Chen, & Chen, 2003; Vincenti, Cassano, Rocchi, & Persico, 1996; Watson, Webb, Bottomley, & Brenchley, 2000). The *VEGF* +936 C/T polymorphism (rs3025039), is a common SNP located in the 3’ untranslated region, and C allele is associated with substantially increased serum VEGF levels when compared to the variant T allele (Krippl et al., 2003; Li et al., 2011; Renner, Kotschan, Hoffmann, Obermayer‐Pietsch, & Pilger, 2000; Watson et al., 2000).

Few studies analyzing *VEFG* on brain tumors have been carried out, although today it is believed that the +936 C/T polymorphism is associated with low‐grade gliomas, being thus a risk factor for progression (Quon et al., 2010). Some studies, however, rule out that this could have an impact on the prognosis of GBMs (Sjöström et al., 2011).

In the present study we assess the frequency of *IDH1* ^*R132H*^ mutation and *VEGF* +936 C/T polymorphism in a Spanish population carrying brain tumors, their association with clinical variables, overall survival (OS) and progression free survival (PFS).

## 2 Material and Methods

### 2.1 Study subjects

The study cohort comprised 80 patients with brain tumors whom underwent surgery at the Department of Neurosurgery, from March 2004 to November 2012. The study was approved by the Hospital Ethics Committee. All patients gave written informed consent, in accordance with the principles established in the 1975 Declaration of Helsinki. Access to medical records and patient data was performed according to protocols established by the Hospital Clínico San Carlos for such purpose.

Patients were divided into two groups whether the tumor was classified as primary or secondary depending on the original histology of the tumor and the evolution to a higher grade glioma in the case of secondary tumors.

On the basis of surgeon's criteria a complete resection was performed on 46 patients (60.5%) and a subtotal resection in 28 patients (36.8%). Tumoral biopsy was done in two patients (2.6%). In four patients the type of surgery could not be determined.

Tumor tissue was obtained by surgical resection and snap frozen in liquid nitrogen in the operating room and stored at −80º until used. Formalin‐fixed paraffin embedded tissues were pathologically examined and classified according to the 2007 WHO criteria. Paired peripheral blood samples were drawn prior to surgery.

After surgical treatment 38 patients (47.5%) and 30 (37.5%) of them underwent adjuvant radiotherapy and chemotherapy, respectively.

### 2.2 Variables studied

Clinical variables included, among others, date of birth, gender, age and date of diagnosis, computed tomography, scan and magnetic resonance imaging (MRI) data, surgery date, complications during surgery, extent of resection, tumor location and pathological diagnosis, radiotherapy‐chemotherapy treatment, pre‐ post‐treatment Karnofsky Performance Score (KPS), and clinical‐radiological follow‐up until last contact or death.

Genetic variables were R132H mutation of *IDH1* gene, in brain tissue, and C/T allele frequency in +936 SNP of *VEGF* gene, in peripheral blood.

OS was calculated as the elapsed time from the date of surgery until death and PFS was calculated from the day of surgery until tumor progression or death. Survival data was obtained from medical history or by direct contact with patients or with their families. If the patient was alive, the date of last follow‐up was used as reference.

DNA from tumor tissue and peripheral blood was extracted using the DNeasy Blood & Tissue Kit (QIAGEN^®^ Hilden, Germany) according to the manufacturer's instructions.

### 2.3 Genotyping of *IDHI* gene mutation

*IDH1* gene mutation in tumor tissue was determined by PCR‐RFLP (Polymerase Chain Reaction‐Restriction Fragment Length Polymorphism), using BclI restriction enzyme (New England Biolabs). Exon 4 of *IDH1* gene was amplified using 100 ng of DNA in a final volume of 25 μl. The amplification mix had 15 pmol of each primer (forward 5′‐GATGGGTAAAACCTATCATCATTGA ‐3′, reverse 5′‐TGTGTTGAGATGGACGCCTA‐ 3′; Meyer et al., 2010), and 20 μl of Platinum ^®^ PCR Supermix High Fidelity (Life technologies) containing polymerase, salts, magnesium, and dNTPs. Amplification was performed with a 55°C annealing temperature for 40 cycles. PCR products were digested in a final volume of 20 μl during 2 hr at 50°C; digestion products were subject to electrophoresis using an 8% polyacrylamide gel.

### 2.4 Genotyping of *VEGF* gene polymorphisms

*VEGF* +936 C/T polymorphism in peripheral blood was determined by TaqMan® MGB probes based polymerase chain reaction. The primers and probes sequences used for PCR were: forward 5′‐ACT CCG GCG GAA GCA TTC‐3′, reverse 5′‐AGC AAG AAA AAT AAA ATG GCG AAT CCA‐3′, probe‐C 5′‐FAM‐CAA GAG GGA CCG TGC TG‐MGB‐NFQ‐3′ and probe T 5′‐TET‐AAG AGG GAC CAT GCT G‐MGB‐NFQ‐3′ (Vidaurreta et al., 2010). Amplification was performed with a 60°C annealing temperature for 40 cycles. PCR was performed in a final volume of 25 μl containing 12.5 μl of Taqman Universal PCR Master Mix (Life technologies) and 10 μmol/L of each primer and probe. The different genotypes were evaluated by quantitative real time PCR in a multiplex reaction using Smart Cycler (CEPHEID, Sunnyvale, CA, USA).

### 2.5 Statistical method

Statistical analysis was performed using MSAccess 2007 software (Microsoft Windows^®^, Redmond, WA, USA).

Quantitative variables were expressed as their mean and range or median and interquartile range if there was asymmetry or elevated dispersion. Qualitative variables were provided with their absolute frequency and relative frequency. Association between qualitative variables was evaluated using the χ² test or the Fisher Exact test when 25% of expected frequencies were lower than 5. There was a statistically significant difference with *p*‐values lower than .05.

Chi‐square tests were conducted to examine whether the genotype frequency of VEGF +936C/T was in Hardy–Weinberg equilibrium (HWE).

Kaplan–Meier curves were obtained to determine and compare OS and PFS among the groups using Breslow's exact test. The hazard ratio (HR) was given with a 95% confidence interval (CI 95%).

## 3 Results

### 3.1 Sample description

The study cohort comprised 80 patients, 73 primary and 7 secondary tumors. The median patient age was 56.5 years old (range 54–65 years). Most of the patients were females, 65% while 35% were males. The clinicopathological variables of patients classified on primary or secondary tumor are summarized in Table 1.

**Table 1:** Clinicopathological variables of the 80 patients with primary and secondary brain tumors

Category	Primary tumor *n* (%)	Secondary tumor *n* (%)	*p*
No patients			
80	73 (91.2)	7 (8.8)	
Age (years)			
≥56: 44 (55.0)	41 (93.2)	3 (6.8)	.49
<56: 36 (45.0)	32 (88.9)	4 (11.1)	
Gender			
Male: 28 (35.0)	25 (89.3)	3 (10.7)	.65
Female: 52 (65.0)	48 (92.3)	4 (7.7)	
Preoperative KPS			
≤70: 18 (22.5)	16 (88.9)	2 (11.1)	.67
>70: 62 (77.5)	57 (91.9)	5 (8.1)	
Type of tumor resection^a^			
Total: 46 (60.5)	42 (91.3)	4 (8.7)	.89
Subtotal: 28 (36.8)	26 (92.8)	2 (7.2)	
Biopsy: 2 (2.6)	2 (100)	0 (0)	
Tumor location			
Supratentorial: 71 (88.8)	64 (90.1)	7 (9.9)	.62
Infratentorial: 7 (8.7)	7 (100)	0 (0)	
Supra‐infratentorial 2 (2.5)	2 (100)	0 (0)	
Postoperative complications			
Yes: 19 (23.7)	18 (94.7)	1 (5.3)	.54
No: 61 (76.3)	55 (90.2)	6 (9.8)	
WHO grade			
I: 21 (26.2)	21 (100)	0 (0)	**<.001**
II: 15 (18.7)	15 (100)	0 (0)	
III: 8 (10)	3 (37.5)	5 (62.5)	
IV: 36 (45)	34 (94.4)	2 (5.6)	
Post‐treatment KPS^b^			
≤70: 18 (23.4)	14 (77.8)	4 (22.2)	**.027**
>70: 59 (76.6)	56 (94.9)	3 (5.1)	

KPS, Karnofsky Performance Score; RT, Radiotherapy; QT, Chemotherapy.

a No data in four patients.

b Post‐surgery or post‐RT/QT KPS: in three cases could not be assessed.

Bold values mean statistically significant relation.

To assess the degree of the resection, a cranial MRI was carried out in 50 patients (62.5%) within the first two days after surgery. It was verified that the resection was complete in 21 patients (42%), and subtotal in 29 cases (58%).

Median time between symptom onset and diagnosis was 15 days, which varied from the incidental finding up to 60 days. Most patients had a pre‐operative KPS over 70 (77.5%), and in 76.6% of the cases (59 patients) a KPS of over 70 was maintained immediately after treatment (postoperative or post radiochemotherapy). In three patients KPS after treatment could not be assessed. Post‐treatment KPS >70 value was related to primary tumor cohort, thus 94.9% of patients harboring a value over 70 were part of this population (*p* = .027).

Taking account of location, 71 tumors (88.8%) were supratentorial and 7 (8.7%) infratentorial. Two tumors (2.5%) had supra and infratentorial location. All secondary tumors were supratentorial in location.

Regarding pathological classification, primary tumor cohort comprised 36 low grade neoplasias, WHO grade I and II, (18 meningiomas, 5 diffuse astrocytomas, 5 ODGs, 3 ependymomas, 2 pilocytic astrocytomas, 2 haemangiopericytomas and 1 haemangioblastoma), 3 WHO grade III (anaplastic astrocytomas) and 34 WHO grade IV (33 GBMs and 1 medulloblastoma). Secondary tumor population was composed of 5 WHO grade III (2 anaplastic oligoastrocytomas, 2 anaplastic astrocytomas and 1 anaplastic ODG) and 2 WHO grade IV, both of them GBMs (Table 2). We observed statistically significant relation between WHO grade and primary and secondary groups (*p* < .001). All low grades were found among primary tumor cohort (Table 1).

**Table 2:** Brain tumors classification in accordance with *IDH1* mutation and *VEGF* polymorphism

Primary tumors	*N*	*IDH1* heterozygote, %	*VEGF* +936 C/T polymorphism^a^, %
Meningioma	18	0	33
Diffuse astrocytoma	5	20	0
Oligodendroglioma	5	100	60
Ependymoma	3	0	33
Pilocytic astrocytoma	2	0	100
Hemangiopericytomas	2	0	0
Hemangioblastoma	1	0	0
Anaplastic astrocytoma	3	0	33
GBM	33	3	12
Medulloblastoma	1	0	0

Secondary tumors	*N*	*IDH1* heterozygote, %	*VEGF* +936 C/T polymorphism^a^, %
Anaplastic oligoastrocytomas	2	50	0
Anaplastic astrocytomas	2	100	0
Anaplastic oligodendroglioma	1	100	100
GBM	2	50	0

GBM, Glioblastoma multiform.

a The CT and TT variants of *VEGF*.

The median follow‐up period was 15 months (463 days; range 5–58 months) and survival analyses were referred to this median follow‐up period. In our patients population the OS at 15 months was 70% (95% CI = 58–80) with 2 years survival of 61.6% and 5 year survival of 48.9%. There were no statistical differences between primary and secondary tumors with respect to OS (70% vs. 75%, *p* = .967). The PFS of primary tumors at 15 months was 51% (95% CI = 38.6–63).

During follow‐up 34 patients died, 32 (94.1%) as a result of progression of disease. This comprises 23 GBMs, 6 grade III astrocytomas, 2 grade II astrocytoma and 1 meningioma. The remaining two patients (5.9%) died due to systemic disease.

Recurrence was detected in 10 patients with primary tumors (13.7%). Eight of them presented disease progression after tumor recurrence.

### 3.2 *IDH1* mutation analysis

*IDH1* ^*R132H*^ mutation was found in 12 patients (15%). It was not possible to analyze the mutation in one case of meningioma due to insufficient tissue sample. In all cases, R132H mutation appeared in heterozygosity. *IDH1* ^*R132H*^ mutation was more frequent in ODGs (100%) than in astrocytic tumors (25%) and in secondary GBMs (50%) over primary GBMs (3%) (Table 2). Neither mutation was found in WHO grade I tumors, meningiomas, medulloblastoma, ependymoma, hemangiopericytomas, hemangioblastoma or in infratentorial tumors. The *IDH1* association with clinical variables is shown on Table 3.

**Table 3:** Relationships of clinicopathological variables with *IDH1* ^*R132H*^ mutation

Category	Primary brain tumor *n* (%)			Secondary brain tumor *n* (%)		
	*IDH1* heterozygote	*IDH1* wild type	*p*	*IDH1* heterozygote	*IDH1* wild type	*p*
Age (years)						
≥56	4 (9.7)	37 (90.3)	.991	2 (66.7)	1 (33.3)	.81
<56	3 (9.7)	28 (90.3)		3 (75)	1 (25)	
Gender						
Male	1 (4)	24 (96)	.242	3 (100)	0 (0)	.15
Female	6 (12.8)	41 (87.2)		2 (50)	2 (50)	
Preoperative KPS						
≤70	0 (0)	16 (100)	.13	2 (100)	0 (0)	.29
>70	7 (12.5)	49 (87.5)		3 (60)	2 (40)	
Tumor location						
Supratentorial	7 (11.1)	56 (88.9)	.59	5 (71.4)	2 (28.6)	**.021**
Infratentorial	0 (0)	7 (100)		0 (0)	0 (0)	
Supra‐infratentorial	0 (0)	2 (100)		0 (0)	0 (0)	
WHO grade						
I	0 (0)	20 (100)	**<.001**	0 (0)	0 (0)	.43
II	6 (40)	9 (60)		0 (0)	0 (0)	
III	0 (0)	3 (100)		4 (80)	1 (20)	
IV	1 (2.9)	33 (97.1)		1 (50)	1 (50)	
Post‐treatment KPS						
≤70	0 (0)	14 (100)	.16	3 (75)	1 (25)	.81
>70	7 (12.7)	48 (87.3)		2 (66.7)	1 (33.3)	

In one tumor sample *IDH1* could not be assessed.

Bold values mean statistically significant relation.

All *IDH1* ^*R132H*^ mutations were detected in supratentorial tumors, and there is a statistical association with the secondary cohort (*p* = .021).

*IDH1* ^*R132H*^ mutation was statistically related to WHO grade in primary tumors, 85.7% of *IDH1* mutated tumors were classified as grade II (*p* < .001) (Table 3), being R132H mutation, significantly more frequent in low grades of primary brain tumors (*p* = .042) (data not shown). *IDH1* ^*R132H*^ mutation shows a different distribution between primary and secondary tumors, R132H mutation was significantly related to secondary tumors, 71.4% of them presented this alteration, in contrast to 9.7% of primary tumors (*p* < .001) (Table 4).

**Table 4:** *IDH1* ^*R132H*^ mutation in accordance with tumor classification

	*IDH1* heterozygote	*IDH1* wild type	*p*
Primary tumor	7 (9.7)	65 (90.3)	<.001
Secondary tumor	5 (71.4)	2 (28.6)	

**Figure 1:**
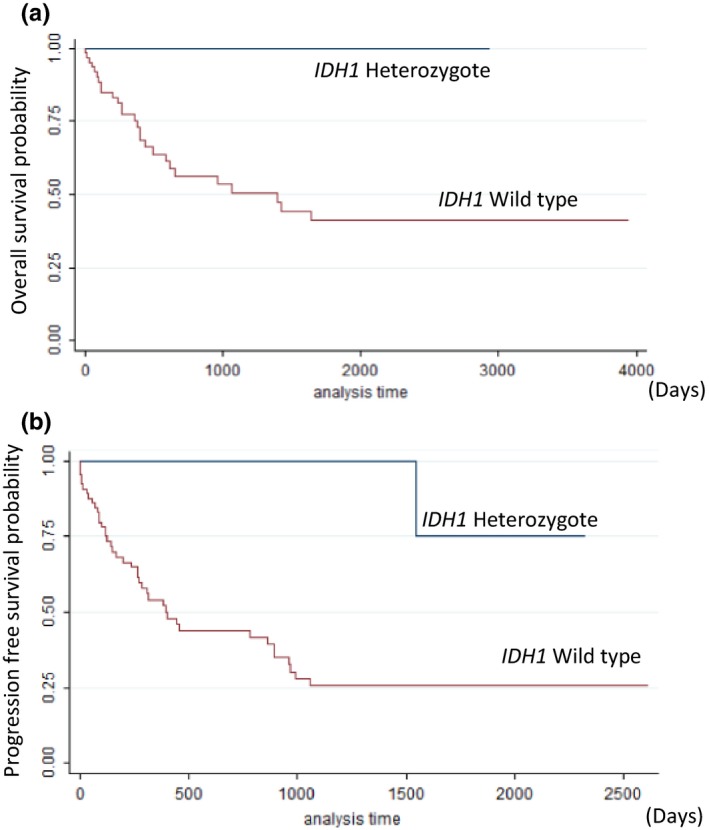
Kaplan–Meier survival curves. Reflects the probability of survival in OS or the probability of having no recurrence (PFS) in relation to the *IDH1* status. (a) *IDH1* OS curve shows survival in days for the mutated variant and for wild‐type variant. (b) *IDH1* PFS curve shows survival in days for mutated variant and for wild‐type variant

In univariate analysis of OS, clinically significant differences were found in OS according to the *IDH1* ^*R132H*^ mutation in primary tumors, the survival after 15 months in patients showing R132H mutation was 100% and 66% in the patients without this mutation (*p* = .059) (Table 5) (Figure 1a). Primary tumor patients with mutated *IDH1* had a relative but not significant OS advantage (HR = 0.35; 95% CI = 0.11–0.15; *p* = .083). Only three patients with *IDH1* ^*R132H*^ mutation died, all of them had secondary tumors, but no significant differences were found between the presence or absence of *IDH1* mutation and OS in secondary tumors.

**Table 5:** *IDH1* and *VEGF* +936 C/T univariate analysis of OS in the 80 patients with brain tumors. Breslow's exact test

Variable	OS (463 days), %	CI95%	*p*
*IDH1*			
Heterozygote	90	47–98	.085
Wild type	66	52–77	
*IDH1* primary			
Heterozygote	100	–	**.059**
Wild type	66	51–77	
*IDH1* secondary			
Heterozygote	80	20–96	.521
Wild type	66	5–94	
*VEGF*			
CC	69	5–94	.627
CT	68	35–86	
TT	66	54–80	
*VEGF* primary			
CC	69	5–94	.663
CT	65	32–85	
TT	66	52–80	
*VEGF* secondary			
CC	71	25–92	.307
CT	100	–	
TT	–	–	

OS, Overall Survival; CI 95%, 95% confidence interval.

Bold values mean statistically significant relation.

In the PFS analysis, none of the patients harboring *IDH1* ^*R132H*^ mutation progressed and 57% of patients without it progressed at 15 months. Significant influence in the PFS was detected for primary tumors in relation to this mutation (*p* = .025) (Figure 1b).

### 3.3 *VEGF* +936 C/T polymorphism analysis

The genotypic distribution of *VEGF* polymorphism in primary tumors did not confirm the HWE (χ^2^ = 4.19, *p* = .04) but secondary tumor samples were in accordance with HWE (χ^2^ = 0.04, *p* = .83).

The *VEGF* +936 T allele was found in the peripheral blood of 16 patients (20%), most of them were primary tumors. The genotype distribution of the *VEGF* SNP and its relation with clinicopathological variables are shown in Table 6. All patients with TT genotype were WHO low grades (*p* = .535) and +936 CC genotype was statistically most frequent in supratentorial location of secondary tumors (*p* < .001). There were no association between +936 C/T SNP and the other clinical variables.

**Table 6:** Relationships of clinicopathological variables with *VEGF* +936 C/T polymorphism

Category	Primary brain tumor *n* (%)				Secondary brain tumor *n* (%)			
	*VEGF* CC	*VEGF* CT	*VEGF* TT	*p*	*VEGF* CC	*VEGF* CT	*VEGF* TT	*p*
Age (years)								
≥56	32 (78)	7 (17.1)	2 (4.9)	.914	2 (66.7)	1 (33.3)	0 (0)	.21
<56	26 (81.2)	5 (15.6)	1 (3.1)		4 (100)	0 (0)	0 (0)	
Gender								
Male	19 (76)	5 (20)	1 (4)	.839	3 (100)	0 (0)	0 (0)	.35
Female	39 (81.3)	7 (14.5)	2 (4.2)		3 (75)	1 (25)	0 (0)	
Preoperative KPS								
≤70	13 (81.3)	3 (18.7)	0 (0)	.63	2 (100)	0 (0)	0 (0)	.49
>70	45 (78.9)	9 (15.8)	3 (5.3)		4 (80)	1 (20)	0 (0)	
Tumor location								
Supratentorial	51 (79.7)	11 (17.2)	2 (3.1)	.63	6 (85.7)	1 (14.3)	0 (0)	**<.001**
Infratentorial	5 (71.4)	1 (14.3)	1 (14.3)		0 (0)	0 (0)	0 (0)	
Supra‐infratentorial	2 (100)	0 (0)	0 (0)		0 (0)	0 (0)	0 (0)	
WHO grade								
I	15 (71.4)	4 (19)	2 (9.5)	.535	0 (0)	0 (0)	0 (0)	.49
II	11 (73.3)	3 (20)	1 (6.7)		0 (0)	0 (0)	0 (0)	
III	2 (66.7)	1 (33.3)	0 (0)		4 (80)	1 (20)	0 (0)	
IV	30 (88.2)	4 (11.7)	0 (0)		2 (100)	0 (0)	0 (0)	
Post‐treatment KPS								
≤70	11 (78.6)	3 (21.4)	0 (0)	.63	4 (100)	0 (0)	0 (0)	.21
>70	44 (78.5)	9 (16.1)	3 (5.4)		2 (66.7)	1 (33.3)	0 (0)	

Bold values mean statistically significant relation.

None of secondary brain tumors presented TT genotype, and only one patient with secondary tumor showed CT genotype. No statistical relation was found in genotypes distribution between primary or secondary tumors (*p* = .840) (Table 7).

**Table 7:** *VEGF* +936 C/T polymorphism genotype distribution based on tumor classification

	CC	CT	TT	*p*
Primary tumor	58 (79.45)	12 (16.44)	3 (4.11)	.840
Secondary tumor	6 (85.7)	1 (14.3)	0 (0)	

In the univariate analysis no significant differences in OS were detected for *VEGF* genotypes. The survival after 15 months in patients showing CC, CT and TT genotypes was 69%, 68% and 66% respectively (*p* = .627). No significant differences were found between the three genotypes of *VEGF* +936 SNP and OS when stratified according to primary or secondary tumor (Table 5). Allele T presence showed a HR = 0.69 (95% CI = 0.30–1.57; *p* = .377).

In the PFS analysis, no significant differences were found between the three genotypes of *VEGF* +936 SNP in primary tumors (*p* = .275). A total of 55%, 44% and 34% of patients with CC, CT and TT genotypes, respectively, progressed at 15 months (data not shown).

## 4 Discussion

Here we report the genetic variables in a Spanish cohort of patients with brain tumors, which presents a distribution of gliomas similar to other studies previously published in other countries (Lewandowska et al., 2014; Mellai et al., 2011; Reuss et al., 2015; Thota et al., 2012) with the exception of the low number of WHO grade III tumors available on this study. From a clinical point of view, there were two clinical parameters that showed differences between primary and secondary cohorts, both related to the better outcome of primary tumors. All low grade gliomas were primary gliomas, whereas in the secondary cohort only higher grades were observed. Moreover post‐treatment KPS > 70 was related to the primary cohort due to the presence of these I and II grade tumors (Table 1).

### 4.1 *IDH1* mutation

We first determined *IDH1* ^*R132H*^ mutation in glioma tumor tissue due to it is the most common missense mutation in *IDH1* gene. Thus, this gene is strongly involved in the biology of gliomagenesis (Ducray et al., 2011; Takano et al., 2012). The *IDH1* gene may be the key in the pathogenic processes associated with the R132H mutation and those caused by the accumulation of R2‐HG (Ducray et al., 2011; Mellai et al., 2011; Parsons et al., 2008; Thota et al., 2012; Weller et al., 2009). This accumulation produces a high intracellular metabolic stress, so cells need to get adapted to this condition to facilitate survival and tumor progression (Van Lith et al., 2014). Previous studies reported a 22–70% of *IDH1* ^*R132H*^ mutation frequency (Ducray et al., 2011; Lewandowska et al., 2014; Mellai et al., 2011; Reuss et al., 2015; Thota et al., 2012). In our study, the frequency of this mutation is lower, 15%; these differences might be due to the presence of a smaller number of patients in WHO grade III primary gliomas and grade IV secondary gliomas. However, R132H mutation distribution is in accordance with previous studies. Mutation has not been described in WHO grade I astrocytoma but appears in 100% of ODGs and not in meningiomas and other primary tumors (Lewandowska et al., 2014; Mellai et al., 2011; Takano et al., 2012; Table 2). Sanson et al. (2009) analyzed the presence of the mutation of *IDH1* ^*R132H*^ based on WHO tumor grading, which was found, from higher to lower frequency, in grade II and grade III astrocytomas and primary GMBs, with similar results found by Ducray and Shibahara groups (Ducray et al., 2011; Shibahara et al., 2011). Our primary tumors cohort presents R132H mutation mainly in grade II group, 86% of heterozygote patients belong to this group, and *IDH1* ^*R132H*^ mutation is related to this stage (Table 3). This finding agrees with the hypothesis of a role of *IDH1* mutation in the early steps of oncogenesis (Juratli et al., 2012; Lewandowska et al., 2014; Thota et al., 2012; Weller, Wick, & von Deimling, 2011).

Within the histological subtypes of GBMs, mutations in *IDH1* are more common in secondary GBMs, fewer than 10% of primary cases and 70–80% of secondary GBMs harbored *IDH1* mutations (Bleeker et al., 2012; Polivka et al., 2014; Takano et al., 2012; Thota et al., 2012) as well as in younger patients (Birner, Toumangelova‐Uzeir, Natchev, & Guentchev, 2011; Ducray et al., 2011; Hartmann et al., 2010; Juratli et al., 2012; Mellai et al., 2011). Our results are coherent with these data, 50% of secondary GBMs, and only 2.9% of primary GMBs carried *IDH1* ^*R132H*^ mutation, however we do not find a significant relationship between mutation and younger patients (Table 3), showing a mean age of 52.6 years old in patients with *IDH1* mutated and 51.8 years old in patients harboring the wild‐type allele. Glioblastoma is the most fatal primary brain cancer (Bleeker et al., 2012), and *IDH1* mutations are frequent in the progressive pathway to secondary GBM (Thota et al., 2012). It is supposed that primary mutated GBMs, are, actually, secondary GBMs with no histological or radiological evidence in the evolution from a less malignant glioma (Bleeker et al., 2012; Hartmann et al., 2010). Several authors suggested that the mutation of *IDH1* could occur in early stages of the glioma formation and could lead to tumor progression towards GBM (Juratli et al., 2012; Lewandowska et al., 2014; Thota et al., 2012; Weller et al., 2011). More than 50% of the total NADPH production in glioblastoma patients is provided by IDH activity and this level is reduced after the occurrence of *IDH1* ^*R132H*^ mutation (Shi et al., 2015) which results in a depletion of intracellular GSH (Ducray et al., 2011; Parsons et al., 2008; Thota et al., 2012), causing oxidative stress and inducing transcription of genes involved in cell death (Kretz‐Remy & Arrigo, 2002). This cellular mechanism permits a slow tumor growth and a better outcome (Lewandowska et al., 2014; Shi et al., 2015).

In an adult population, regarding to location, most gliomas are supratentorial and only 5% or less of diffuse gliomas appear infratentorially (Rineer, Schreiber, Choi, & Rotman, 2010). In this cohort, almost 90% of tumors were located on the supratentorial region and all IDH mutations appeared in this compartment. Moreover, *IDH1* ^*R132H*^ mutation is related to this location in secondary tumors (Tables 1 and 3). This event could suggest that adult infratentorial gliomas arise through a tumorigenic pathway distinct from supratentorial gliomas (Miwa et al., 2009; Sano et al., 2013), and even they could be two different histological entities (Miwa et al., 2009). In fact, most of published data analyze supratentorial gliomas because of their high frequency. Adult infratentorial gliomas have not been genetically well assessed. In this study no *IDH1* mutation was found in infratentorial tumors, this data agree with Yao et al. (2013), although Ellezam et al. (2012) found 7% positive tumors for the *IDH1* ^*R132H*^ mutation by immunohistochemistry in grade II and III brainstem diffuse gliomas.

Regarding the survival analysis, previous studies demonstrated the prognostic significance of *IDH1* mutations (Arita et al., 2015; Brennan et al., 2013; Ducray et al., 2011; Hartmann et al., 2010; Killela et al., 2014; Lewandowska et al., 2014; Mellai et al., 2011; Olar et al., 2012; Parsons et al., 2008; Polivka et al., 2014; Sanson et al., 2009; Shibahara et al., 2011; Sun et al., 2013; Takano et al., 2012; Weller et al., 2009). Some authors observed that OS and PFS in IDH mutated cases were about twice longer than in wild‐type patients (Arita et al., 2015; Polivka et al., 2014), and others showed that mutation in *IDH1* was an independent factor for a favorable prognosis (Brennan et al., 2013; Ducray et al., 2011; Polivka et al., 2014; Sanson et al., 2009; Shibahara et al., 2011). However, this hypothesis was rejected by other groups taking into account other factors such as surgery and radiotherapy‐chemotherapy treatments when evaluating the survival of these patients (Ichimura et al., 2009; Rineer et al., 2010; Thota et al., 2012). In this Spanish population, patients with primary tumors harboring R132H mutation had a relative but not significant reduction in death risk of 65% compared with the wild‐type patients, and none of these *IDH1* ^*R132H*^ heterozygote patients progressed, conferring this alteration with a significant PFS advantage. The overall survival curve for *IDH1* displayed a trend toward greater survival in the group with the *IDH1* mutation, although the sample size is not sufficient for a statistically significant difference. The 0.35 HR value supports that the presence of the mutation acts as a protecting factor against mortality. In secondary tumors, this effect in survival has not been observed due to the smaller size of this cohort and few cases of secondary GBMs (Table 5 and Figure 1a). The last meta‐analysis published of 55 observational studies and 9487 patients with gliomas showed a significant OS and PFS advantage for patients with *IDH1*/2 mutations over those without them (Xia et al., 2015). This study observed a risk reduction of death of 61%, consistent with our results, and a statistically significant HR due to the large number of individual studies analyzed. These data seems to indicate that biology of wild‐type *IDH1* and mutated *IDH1* ^*R132H*^ is different. When *IDH1* gene is mutated, the α‐KG is not generated, and the α‐KG‐dependent dioxygenases are inactive. These dioxygenases are thought to be involved in epigenetic control, suggesting that mutations in *IDH1* can affect a large number of genes (Watanabe, Nobusawa, Kleihues, & Ohgaki, 2009; Zhao et al., 2009). *IDH1* mutations are likely to be a direct cancer driver in early stage of gliomagenesis promoting extensive alteration of the epigenetic pattern (Turcan et al., 2012; Watanabe et al., 2009).

### 4.2 *VEGF* +936 C/T polymorphism

The *VEGF* +936 C/T polymorphism was the second genetic parameter analyzed in this study. This SNP had been analyzed in several tumors: ovarian (Rinck‐Junior et al., 2015), breast (Jakubowska et al., 2008; Krippl et al., 2003; Rodrigues et al., 2012), lung (Lee et al., 2005), and also in brain tumors (Jiang, Lian, Xie, Li, & Wang, 2013; Li et al., 2011; Sjöström et al., 2011). VEGF is a key factor in angiogenesis, in progression of malignant tumors, increase in vascular permeability and hypercoagulability. It develops its effect through the VEGFR2 receptor, which plays an essential role in the development, prognosis and response to adjuvant therapy of the GBM (Ku et al., 2005). Several studies associate brain tumors with high *VEGF2* expression. Quon et al. (2010) established this relation with ODG, especially with anaplastic grade, where the presence of this alteration conferred a greater risk for tumor progression and poor prognosis. In ependymomas, the association was also demonstrated by Korshunov, Golanov, and Timirgaz (2000). Inhibition of VEGF activity is able to reduce angiogenesis and tumor growth (Jiang et al., 2013). *VEGF* +936 C/T polymorphism correlated with lower levels of the protein, but the mechanism by which this SNP causes the decreased VEGF plasma levels is not established. The presence of T allele has been related with the loss of a potential binding site for activator protein 4 (AP‐4). This may be the mechanism of variant allele for the inhibition of *VEGF* transcription, and explain the lower risk of T carriers in developing cancer (Renner et al., 2000).

The frequency of T allele in the present study was 0.12. This data is in agreement with those published by NCBI dbSNP database for European population (minor allele frequency, MAF, of 0.13) and comparable to previous results from Caucasian and European cohorts (frequency 0.13–0.16; Renner et al., 2000; Rinck‐Junior et al., 2015; Rodrigues et al., 2012; Vidaurreta et al., 2010), but quite different to Korean and Chinese cohorts (frequency 0.16–0.24; Jiang et al., 2013; Lee et al., 2005; Li et al., 2011) which present a MAF of 0.18 (NCBI dbSNP database; Li et al., 2011). In our study, T allele appears more frequently in pilocytic astrocytomas and oligodendroglial tumors, followed by meningiomas, ependymomas and anaplastic astrocytomas in frequency (Table 2). Homozygote variant carriers only appeared in early stages, but we did not find significant relationship between genotype distribution and tumor WHO stage in primary tumors (Tables 6 and 7). *VEGF* +936 C/T SNP is not related to clinical aspects of gliomas in this Spanish population. Similarly, Quon et al. (2010) detected a high VEGF protein expression in ODG cells and related higher expression with WHO grade III, although these authors didn't study the genetic status. The association of VEGF increased expression with advanced stages can be explained by its role in promoting endothelial proliferation, migration and organization into functional vessels (Hicklin & Ellis, 2005).

Several studies in Chinese populations showed TT genotypes associated to glioma patients when compared to control subjects (Jiang et al., 2013; Li et al., 2011), and this genotype was also related to glioma grade IV and GBMs in a Han Chinese cohort (Jiang et al., 2013). Given that frequencies of a single polymorphism are quite different between ethnic populations, the effect of this SNP on cancer susceptibility could vary. Yang et al. (2011) performed subgroup analysis for Caucasian and Asian studies and could not show association between the T allele and breast cancer risk. However, Rodrigues et al. (2012) found a protective association between T allele carriers and breast cancer in a Spanish population. Other works have reported a significantly protective effect of the CT/TT genotypes on breast cancer (Jakubowska et al., 2008; Krippl et al., 2003) and ovarian tumors (Rinck‐Junior et al., 2015) in Caucasian populations. Sjöström et al. (2011) focused their study on GBMs, concluding that the polymorphic variations of *VEGF* didn't impact on the prognosis of these tumors, compared to the variations in *VEGFR2*, its receptor. Our data are consistent with this investigation, since no statistically difference for survival in association with the SNP was found, although the unique secondary tumor that presents T allele remains alive at 15 months while CC secondary tumors presented an OS of 71% (Table 5). In primary tumors, CC patients had a lower but no statistically significant PFS, suggesting that a longer clinical follow up of these CT and TT patients, could show a better outcome in this subgroup due to a lower VEGF levels. This hypothesis is consistent with the protective association between the +936 T carriers and cancer showed by Krippl et al. (2003), and with the theory of Quon et al. (2010) suggesting that VEGF high expression constitutes an early marker for identifying a group at high risk for tumor progression. Higher levels of VEGF, related to CC genotype, could promote stimulation of the angiogenesis helping the tumor to become more aggressive (Yang et al., 2011).

## 5 Conclusions

*IDH1* ^*R132H*^ mutation is related to WHO grade II in primary brain tumors and shows high frequency in ODGs and in secondary GBMs. R132H mutation has been associated with the prognosis of patients with primary brain tumors; mutation carriers showed better PFS and a tendency towards a higher survival has also been observed. These are promising results and should be confirmed on larger cohorts of primary tumors.

The *VEGF* +936 C/T polymorphism is more common in oligodendroglial tumors but it is not related to any clinical variable of tumor. There was no relation between the presence of this polymorphism, the risk of tumoral progression and survival.

## 6 Limitations

Main limitation of this study depends on the heterogeneous series of brain tumors that we have considered. Another limitation is related to the small number of cases in comparison with the number of tumor types taken into account. Both of them can explain why no clear‐cut results come out from our paper.

## Conflict of Interest

The authors declare that they have no conflict of interest.
